# Toward making the field talk: assessing the relationship between digital technology and sustainable food production in agricultural regions

**DOI:** 10.3389/fnut.2024.1462438

**Published:** 2024-11-07

**Authors:** Nawab Khan, Xuanguo Xu, Muhammad Khayyam, Abdul Raziq

**Affiliations:** ^1^College of Economics and Management, Shandong Agricultural University, Tai’an, China; ^2^College of Public Administration, Shandong Agricultural University, Tai’an, China; ^3^School of Economics and Management, Southwest Jiaotong University, Chengdu, China; ^4^Directorate of Vegetable and Seed Production Agriculture Research Institute, Village Aid Sariab, Quetta, Pakistan

**Keywords:** food systems, sustainability, crises, technology, PSM, climate change

## Abstract

**Introduction:**

The global food system faces numerous challenges, including population growth, pandemics, climate change, natural disasters, and economic instability. These challenges have a profound impact on agriculture, with climate change leading to unpredictable weather and more frequent extreme events that threaten crop yields and farming sustainability. Farmers are also grappling with rising input costs and market volatility, intensifying the need for improved productivity and efficiency.

**Purpose:**

Considering these challenges, digital technology, particularly mobile internet (MI), is emerging as a key tool for achieving sustainable agriculture by enhancing productivity, efficiency, and cost-effectiveness. While much research has focused on the effects of MI on agricultural inputs, prices, and operational efficiency, there is limited understanding of its direct impact on food production outcomes. This study aims to fill this gap by examining the influence of MI usage on crop production.

**Method:**

The study utilizes data from 660 farmers across two provinces of Pakistan. Propensity Score Matching (PSM) and linear regression models are employed to assess the impact of MI usage on food production.

**Results:**

The analysis reveals a significant positive effect of MI usage on crop production, with MI users experiencing a 13.30% increase in crop yield compared to non-users. The heterogeneity analysis shows varying impacts among different farmer groups: young farmers see a 13.50% increase, less-educated farmers a 15.27% rise, larger-scale farmers a 23.80% boost, and those in economically developed villages a 10.50% improvement per hectare compared to non-users.

**Conclusion:**

The study highlights the potential of MI in boosting crop production, particularly for specific farmer demographics. Policymakers should prioritize the development of MI infrastructure in rural areas and collaborate with research institutions, agricultural cooperatives, and enterprises to design interventions, such as financial support and technical assistance, that can help farmers fully leverage the benefits of digital technology.

## 1 Introduction

Increasing agricultural productivity and safeguarding food resources are crucial for global efforts and are integral to sustainable national development (1). The outbreak of the COVID-19 pandemic has exacerbated global food security challenges and underscored the important role of food as a stabilizing force (2). To enhance global food security, there is an urgent need to strengthen domestic production (3). Agriculture sector serves as the backbone of the global economy providing income, and employment services, particularly in remote sides with vast arable land (4). In many emerging economies, agriculture is a fundamental sector that not only meets the needs of a growing population but also provides essential raw materials and supports manufacturing and service industries (5). It plays a key role in a complex network of input–output value chains and serves as a repository of surplus labor from other industries and services. Smallholder agriculture has become a potent force in low-income and emerging nations striving for food security and livelihoods (6). Although these small farms occupy only 12% of the globe’s arable land, they produce 80% of the food in regions such as Asia and sub-Saharan Africa (6). Improving production in the agricultural sector holds great promise for eradicating poverty, enhancing nutrition and food security at all levels of society, and advancing the ambitious agenda of sustainable development goals (SDGs) of the United Nations.

Furthermore, growers in certain developing nations encounter notable obstacles in acquiring essential market information, knowledge, and skills necessary for boosting their incomes (5). These hurdles encompass information disparities between import traders and export customers, escalating transaction expenses, inadequate agricultural services, and restricted access to credit facilities (7). Of significant concern is the pervasive information asymmetry that inhibits farmers, particularly those residing in rural areas, from adopting transformative inputs like information communication technology (ICT) and mobile internet (MI) to enhance yields (8). Consequently, these farmers contend with diminished crop yields and income, thwarting their livelihood and impeding rural advancement (9). Hence, the implementation of advanced approaches to alleviate information asymmetries, particularly through leveraging MI to enhance farm performance, holds considerable promise (4).

Harnessing digital technologies can be pivotal in alleviating information asymmetries by enabling swift and cost-effective dissemination of information. Current evidence suggests that adopting MI could enhance smallholder growers’ access to financial and agricultural services (10), as well as facilitate access to input and output markets (11, 12), thereby fostering income-generating activities like off-farm pursuits and agricultural marketing. Recognizing the immense benefits of leveraging MI, numerous countries have initiated various MI-driven initiatives aimed at enhancing farm performance and fostering rural progress (13). For instance, China’s Internet-centric agenda encompasses frameworks such as internet plus agriculture plus finance, farmer field schools, and rural e-commerce (14). Moreover, a plethora of studies have underscored the positive impact of utilizing ICT such as computers, mobile, and internet technology on agricultural and rural advancement (14). However, these findings delve into the intricacies of ICT adoption, addressing selection bias through methodologies like instrumental variables (IV), PSM, and endogenous treatment regression (ETR) models. Through the utilization of PSM modeling, Issahaku et al. (15) demonstrated that MI use significantly enhanced agricultural development in Ghana. These findings underscore the transformative potential of digital technologies in empowering smallholders and propelling rural advancement.

The adoption of agricultural technologies can significantly influence crop production by shaping grower practices and resource utilization, including capital assets, labor, pesticides, and fertilizers. Technical efficiency, a crucial metric, reflects the ratio of actual yield achieved by growers to the maximum attainable yield given the inputs utilized (16, 17), thus indicating the efficiency of agricultural input utilization. Existing literature underscores the substantial impact of ICT adoption on grower behavior concerning fertilizers and seeds practice (18), besides land growth (19). Notably, ICT adoption has been shown to influence grower decisions regarding fertilizers and seeds usage and land expansion. However, despite the importance of ICT, few studies have specifically examined its impact on crop production. A study conducted by Mwalupaso et al. (19), which investigated the influence of mobile technology access to information on maize productivity in Zambia. The findings reveal that grower technical efficiency significantly improves with mobile phone utilization, underscoring the potential of ICT adoption to enhance agricultural productivity and efficiency, particularly in areas with limited information access.

While these findings may not lead to uniform assumptions about MI adoption, the descriptive variables employed play a pivotal role in elucidating the factors influencing farmers’ decisions to adopt MI. Furthermore, the aggregation of these insights can serve as a valuable asset for policymakers and agri-extension officers, aiding in the strategic dissemination and enhancement of MI technology. Nevertheless, notable gaps persist in the evidence concerning the impact of ICT on welfare in Pakistan. Limited investigations have explored ICT acceptance and efficacy, and there has been no concerted effort to assess ICT adoption and its implications for wheat productivity in the country. Consequently, this article underscores the imperative of fostering digital agricultural informatization. By comprehensively understanding the key determinants of MI adoption, we can effectively and efficiently pursue our objectives of enhancing agricultural productivity. This entails not only addressing existing gaps in research but also prioritizing efforts to cultivate a conducive environment for the uptake of digital technologies in agriculture.

Despite the growing importance of digital tools in agriculture, there remains a lack of studies assessing the effects of MI usage on grain production, especially from the viewpoint of farmers. In this context, digital technology refers to farmers’ use of MI for accessing key agricultural data, including information on input quantities and prices, production techniques, and market dynamics. By analyzing household survey data, this article explores the influence of MI on agricultural productivity and aims to fill a gap in the literature concerning agricultural markets and policy frameworks. The primary objectives of the current research are twofold, firstly, to explore whether and to what extent the adoption of MI influences crop production, and secondly, to examine the effects of agricultural mechanization utilization on crop production across farmers with varying ages, education levels, farm sizes, and levels of economic development in their respective areas, while considering potential idiosyncratic effects.

The paper follows this structure: Section 2 reviews pertinent literature, Section 3 outlines the methodology, Section 4 presents the findings and discussion, and Section 5 offers the conclusion, policy implications and discusses limitations.

## 2 Review of literature

In recent decades, ICT has undergone significant advancements across various domains. Given its potential to catalyze economic and societal transformations, extensive research has been conducted to assess its impacts on various fronts (20). Initially, research primarily focused on production, economic growth, and poverty alleviation, with ICT being regarded as a constituent element of the production function alongside land, capital, and labor (20). A multitude of studies have indicated that ICT exerts a positive influence on employment (21), household income, and labor mobility (22). Some scholars argue that ICT holds the potential to reshape rural economies and narrow global development disparities (23). Furthermore, research on ICT has increased to encompass diverse topics, including bridging the gender gap (24), fostering entrepreneurship (25), and enhancing financial empowerment. ICT facilitates advancements in these areas by augmenting the efficiency of information generation, transmission, and access, reducing search and transaction costs, and enabling more streamlined production and management systems.

Numerous factors affecting agricultural productivity have been known (15). Over the past two decades, a substantial past study has underscored the significance of ICT (26). Initial evidence by Lio and Liu (27) demonstrated the role of ICT in augmenting agricultural production across 81 countries between 1995 and 2000. The following studies by numerous scholars corroborated these findings. For instance, Qgutu et al. (28) illustrated that the widespread adoption of ICT enhances the productivity of small-scale agriculture by addressing information asymmetry issues. Additionally, internet connectivity has notably supported food production in Vietnam (13) and alleviated poverty in rural areas (29). Similarly, research performed in Pakistan revealed that the utilization of mobile phone and the Internet augmented the income of wheat growers, indicating enhanced marketing and sales efficiency, thereby increasing crop profitability (30). Deng et al. (31) further stated that internet usage improves resource efficiency and diminishes agricultural waste.

A substantial studies evidence corroborates the pivotal role of the Internet in agricultural production, prompting inquiries into its underlying causes. These investigations underscore the importance of augmenting human capital and facilitating access to information. Access to agricultural technology information aids growers in diversifying crops, optimizing land and input allocation (32), and expanding land holdings (19), thereby enhancing productivity. Furthermore, addressing challenges such as knowledge asymmetry and adverse selection assists farmers in making informed decisions and implementing more effective management practices. Facilitating farmer communication and offering learning opportunities can significantly enhance social capital and information literacy, consequently influencing farmers’ adoption of more productive agricultural techniques. For instance, farmers with access to internet-based resources tend to exhibit greater proficiency in utilizing chemical inputs such as fertilizers and pesticides. Additionally, information technology platforms have the potential to foster family social capital and create an environment conducive to the dissemination and application of production technologies (33). Similarly, Deng et al. (34) highlighted that internet usage influences the perceptions of ecological pollution among rural Chinese growers, suggesting that internet access can serve as a valuable tool in promoting environmentally friendly agricultural development and mitigating ecological issues.

Additionally, the study explores the agricultural industry from many perceptions and assesses the influence of technological progressions on the agricultural sector and farmers’ incomes. For example, a study indicates that the ICT usage can effectively mitigate income inequality in rural areas (34). Moreover, Min et al. (35), utilizing empirical data from 2008 to 2015, concluded that ICT plays a significant role in driving economic expansion and growth. The hypothesis that the adoption of information technology positively influences the well-being of rural growers finds support in the work of Ma et al. (23), with Nie et al. (36) reinforcing these conclusions. Existing literature generally concurs that technology yields beneficial effects on agriculture. Building upon these findings, this study specifically investigates the impact of information technology on wheat crop yield. While some studies focus on various crops in Pakistan, most explore the long-term implications of climate change. Research canter on crop production differs from this approach in several fundamental aspects. Recent work by Lin et al. (37) aligns closely with this study’s thematic focus. Although investigators have examined key factors for enhancing agricultural yields, unlike current findings, they place greater emphasis on collaborative engagement.

The findings suggest that collaborative contributions supportively influence the overall factor efficiency of SMEs. Specifically, in Pakistan, there is a scarcity of studies examining the impact of ICT use on crop yields, especially those focusing on the agricultural sector. Gathering information in this regard will facilitate an understanding of how the agricultural sector, which is grappling with supply and demand pressures stemming from population growth and climate change, can effectively address these issues.

## 3 Methodology

### 3.1 Study areas description, data collection, and variable selection

#### 3.1.1 Study areas description

Khyber Pakhtunkhwa (KP) and Balochistan are distinct provinces within Pakistan, each characterized by unique topography and features. KP, previously known as the North-West Frontier Province, is situated in the northwest region of Pakistan. Its landscape is predominantly hilly, with the Hindu Kush Mountains dominating the western section of the province. While KP is known for its breathtaking landscapes, it is also recognized as an important agricultural region. The province contributes a significant portion to the country’s total cultivated area and plays a vital role in cereal production. In contrast, Balochistan, located in the southwestern part of Pakistan, is the largest province by land area but remains sparsely populated. It boasts diverse landscapes, abundant natural resources, and a rich cultural heritage.

It stands as the largest province in terms of land area but is sparsely populated. Balochistan is endowed with diverse landscapes, unique natural resources, and rich cultural diversity. The province is picturesque, and fertile and plays an important role in tourism and agriculture. Furthermore, Balochistan’s strategic location along the border areas also adds to its importance. Overall, KP and Balochistan are integral provinces of Pakistan and each province in its own way contributes to the country’s agricultural sector, tourism, and strategic importance.

#### 3.1.2 Data collection

**Figure 1:**
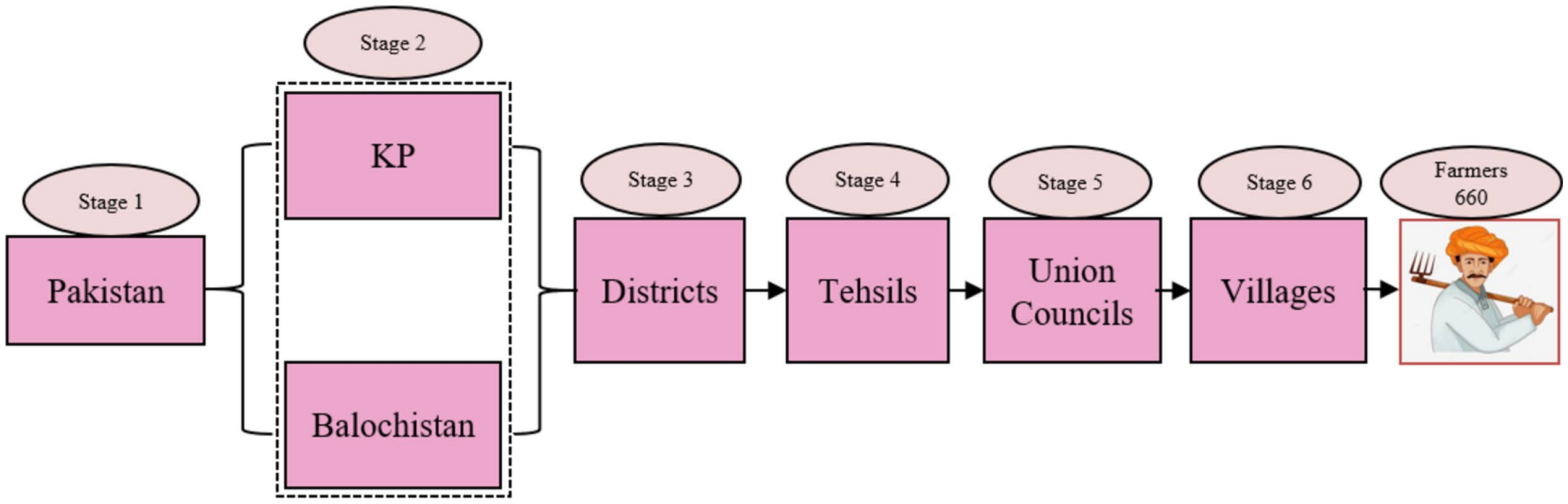
Sampling distribution across study areas.

The current study, conducted in 2023, focused on the KP and Balochistan province in Pakistan. However, 660 questionnaires were distributed to farmers using multistage random sampling techniques to collect data. The objective was to ascertain the impact of MI usage on crop production. The study progressed through seven phases: Pakistan was chosen in the first phase, and KP and Balochistan became the main study area in the second phase. In the third phase, study data were categorized into five districts based on the proportion of agricultural production. The fourth phase involved choosing 10 tehsils from the five districts to administer a predetermined questionnaire. In the fifth phase, 20 UCs were nominated from the selected tehsils. The sixth phase randomly monitored 12 villages from these UCs, involving 660 farmers in the seventh phase (Figure 1).

This study collected data from wheat farmers through interviews and questionnaires. Given the complexity of the questionnaire, we supplemented it with in-depth interviews to ensure a thorough understanding. To enhance reliability, the questionnaire was pre-tested before the main data collection phase. It covered a wide range of information, including the farmers’ socioeconomic profiles, MI usage, and other relevant variables aligned with the research objectives. The gathered data was then carefully edited and coded using Stata 14 software, ensuring uniformity, validity, consistency, completeness, and accuracy. This rigorous process provided a solid foundation for subsequent analysis and interpretation. Furthermore, this method allowed us to capture nuanced insights into the factors influencing MI adoption and its impact on agricultural practices. The detailed data preparation also minimized the risk of bias or data inconsistencies, further strengthening the reliability of the findings.

The representative sample sizes mentioned above were determined using the sample size calculation formula Yamane (38), renowned for its suitability in homogeneous populations (Equation 1). Below is the formula along with the corresponding number of demonstrative samples obtained:


(1) $$n=\frac{N}{1+N\left(e^{2}\right)}\to n=\frac{24,100}{1+24,100\;{\left(0.05\right)}^{2}}\to \frac{24,100}{61.25}=660$$

Among these parameters: “*n*” denotes the required sample size, “*N*” stands for the population size or total number of growers, and “*e*” represents the precision level, with the standard assumption being 5%.

#### 3.1.3 Variable selection

For the current study, we have selected household crop yield as the dependent variable, expressed as household wheat yield per hectare. To derive this, we separated the farmer’s total wheat production in 2023 by the farmer’s wheat sowing area. Apart from MI usage, this study incorporates several control variables known to influence growers’ food production, as evidenced by the research of Yang et al. (39). Growers’ characteristics encompass factors such as education, age, training, health, and risk preference. Household attributes, influenced by Boz’s (40), consist of variables like the proportion of non-farm income, farm size, number of plots, livestock, extension, and market distance. Production inputs, as highlighted by Janssen and Bert (41), encompass costs related to seeds, pesticides, fertilizers, irrigation, machinery, and labor. Additionally, village characteristics, as identified by Tatlıdil et al. (42), comprise main indicators such as village poverty and economic development level. Detailed statistical descriptions of these variables are presented in Table 1.

**Table 1:** Variables descriptive statistics.

Variables name	Description
Wheat production	Wheat production (kg/ha)
Age	Age of the farmers (years)
Education	Level of education attained: Illiteracy = 1; Primary school = 2; High school = 3; College or higher = 4
Plots	Number of Agri-plots owned or managed by the household
Farm size	Size of the farm (ha)
Non-agri-income	Proportion of income from non-agricultural source (PKR)
Livestock	Livestock of farmers (Numbers)
Training	Whether farmers receive training: 1 if yes, 0 if no
Extension	Extension contacts *per annum* (Numbers)
Health	Health status: Good = 1; Normal = 2; Poor = 3; No-labor capacity = 4
Machinery	Charges of machinery (PKR)
Irrigation	Charges of electricity and irrigation (PKR)
Seeds	Charges of seeds (PKR)
Fertilizer	Charges of fertilizers, both chemical and organic (PKR)
Pesticide	Charges of pesticides (PKR)
Risk preference	Types of risk preference: Conservative = 1; Neutral = 2; Preference = 3
Market distance	Distance to the market (km)
Economic development	Level of economic development in the village: Good =1; Better = 2; General = 3; Poor = 4; Very poor = 5
Poor village	Indicator: 1 if the village is classified as poor, 0 otherwise

### 3.2 Theoretical model

#### 3.2.1 Theoretical analysis

Conventional models of farmers’ production behavior often operate under classical theoretical assumptions, assuming that farmers have access to complete information and that markets function efficiently. However, in reality, farmers often struggle to access comprehensive information when making production decisions. Embracing information can alleviate this challenge by reducing farmers’ information costs and providing them with enhanced insights into agricultural technology, market dynamics, and management strategies, thereby facilitating the optimal allocation of resources. The influence of agricultural information on production can be delineated into four primary aspects. Firstly, leveraging MI platforms enables farmers to access a wealth of agricultural production information across various stages. For instance, during the planting phase, farmers can utilize MI to access weather forecasts, seed varieties, and fertilization techniques, aiding in selecting the optimal planting time and applying fertilizers judiciously. In the growth stage, MI offers insights into agricultural diseases and pest management, enabling farmers to effectively prevent and control infestations. As harvest approaches, timely updates on weather conditions and crop readiness via MI empower farmers to make informed decisions about when to harvest, thereby minimizing crop losses (43, 44).

Secondly, the utilization of MI platforms facilitates the adoption of innovative technologies. By providing growers with a deeper understanding of the “risk–reward” dynamics related to new technologies, MI encourages them to embrace innovation while mitigating potential risks (45). Additionally, the integration of MI into agricultural practices promotes increased investment in production and enhances the efficient allocation of agricultural resources (46). Throughout the entire agricultural production process, MI aids in optimizing land, capital, and labor allocation, thereby reducing costs, enhancing productivity, and incentivizing further investment in agricultural endeavors (19, 47). Furthermore, the implementation of MI reshapes farmers’ old-style production paradigms, exposing them to up-to-date management perceptions and fostering a spirit of innovation and adaptability (48). By facilitating access to management information, MI empowers growers to attain novel managerial knowledge and refine their skills. Besides, MI contributes to enhancing farmers’ environmental awareness and stewardship by improving their perception, knowledge, and practices related to environmental sustainability (49).

Created on the insights gained from the previous theoretical study, we developed a conceptual model to provide an in-depth study of the impact of MI adoption on crop yields. The core of farmers’ economic considerations is the pursuit of profit maximization. Therefore, this study design a farmers’ output Equation 2 (denoted as Y ) to explain this phenomenon:


(2) $$Y=A.F\;\left(\mathrm{K,L,S}\right)$$

In this Equation 3, Y represents output; A denotes technological input; K,L,andS signify capital input, labor input, and land input, correspondingly.

Divide both sides by the land areas S :


(3) $$Y=A\;f\left(k,l\right)$$

Where $y=\frac{Y}{S},\;k=\frac{K}{S},\;l=\frac{L}{S}\text{.}$

Equation 3 assumes homogeneity among crop farmers. To address the constraints arising from this assumption, we introduce the characteristics of farmers, families, villages, and regions in the empirical analysis to account for heterogeneity. Additionally, introduce a pivotal variable MI usage. This transformation leads to Equation 4:


(4) $$y=A\;f\;\left(k,l,y,\mathrm{region},MI\right)$$

In the equation, *γ* represents a collection of control variables encompassing farmers’ characteristics, families, and village attributes; region signifies regional attributes; while *MI* denotes the utilization of mobile internet.

The peculiar Equation 5 is as follows:


(5) $$Crop_{i}=\beta_{0}+\beta_{1}MI_{i}+\psi z_{i}+\mathrm{\delta regio}n_{i}+\varepsilon_{i}$$

Here, $Crop_{i}$ denotes the yield of crops produced by a specific farmer, while $MI_{i}$ stands for the household’s utilization of MI, serving as a core variable. The coefficient $\beta_{1}$ signifies the impact of MI usage on crop yield. Control variables influencing crop yield are represented by $z_{i}$ , with ψ representing the coefficients corresponding to these variables. Additionally, regional factors are accounted for through the inclusion of a regional dummy variable $\mathrm{regio}n_{i}$ , with δ representing its coefficient. The term $\varepsilon_{i}$ denotes the error term, capturing unexplained variance in crop yield. This model enables the examination of how MI usage, alongside other control variables and regional characteristics, affects crop productivity among individual farmers.

#### 3.2.2 PSM technique

Considering the varying early situations among growers utilizing and not utilizing MI, for instance, age, level of education, and risk preference, direct regression might introduce selection bias. To mitigate this, PSM establishes a “counterfactual” framework by generating control group outcomes akin to the treatment group. The approach minimizes the risk of sample selection bias (50). The distinct stages of PSM implementation are outlined below:

Initially, this analysis utilizes a logit model to predict the likelihood of growers adopting MI and to assess the propensity score, represented by Equation 6:


(6) $$P\left(X_{i}\right)=F\left(MI_{i}=1|X_{i}\right)=\frac{1}{1+e^{{\_}_{X_{i}B}}}$$

Equation 6 describes the probability $P\left(X_{i}\right)$ of farmer 𝑖 utilizing the agricultural practice MI , where $X_{i}$ represents the influencing factor affecting the farmer’s decision, and *B* is the coefficient associated with this factor. The logistic function F ensures the probability remains within the range of 0 to 1, facilitating effective modeling of the decision-making process.

Additionally, to address the issue of self-selection, various matching methods such as nearest neighbor matching (NNM), radius matching (RM), kernel matching (KM), and local linear regression matching (LLRM) are employed. These methods are utilized to create treatment and control groups, thereby mitigating the problem of self-selection (51).

The NNM relies on estimating propensity scores using a Logit model. The goal is to find control group samples with propensity scores closest to those of the treatment group. Assume denote *C*(*i*) as the set of samples matching the *i*-th sample in the treatment group. The propensity score for each sample is denoted by *p_i_*. The detailed Equation 7 for NNM is as follows:


(7) $$C\left(i\right)=|j\min\;p_{i}-p_{j}|$$

The RM: to address the potential loss of comparability, the RM method is employed. This approach limits the absolute difference between propensity score values to mitigate the risk of matching to distant or dissimilar units. Specifically, we constrain the absolute distance between propensity score values, ensuring that $\left|p_{i}-p_{j}\right|$ is no greater than 0.25*σ*, where 𝜎 represents the sample standard deviation of the propensity score.

The KM: While NNM and RM both fall under the umbrella of NNM, KM introduces a more sophisticated weighting scheme. In NNM, a simple arithmetic average of NNM samples is computed. However, KM assigns weights to individual matches based on their distances. Closer matches are assigned higher weights, gradually tapering off to zero beyond a certain distance threshold (52).

We are exploring the construction of a non-parametric univariate regression model.


(8) $$y_{i}=m\left(x_{i}\right)+\varepsilon_{i}\varepsilon_{i}∼iid\;\left(0,\sigma_{\varepsilon }^{2}\right)$$

In this context Equation 8, *m*(·) represents an unknown function, and $\varepsilon_{i}$ denotes a random error term.

Assuming a specific value $x_{0}$ , for example $x_{0}$ , if $y_{i}$ represents the observed value corresponding to the NNM of $x_{0}$ , then $Y_{0}$ is the “local weighted average estimator in Equation 9.” This estimator calculates the weighted average of the observed values corresponding to the NNM $x_{0}$ .


(9) $$Y_{0}=\overset{n}{\underset{i-1}{\sum }}w\left(i,j\right)y_{i}$$

The weight $w\left(i,j\right)$ in this context is determined by the kernel equation, expressed in Equation 10 as follows:


(10) $$w\left(i,j\right)=\frac{K\left[\left(x_{j}-x_{i}\right)h\right]}{\sum_{i=1}^{n}K\left[\left(x_{j}-x_{i}\right)h\right]}$$

In this context, ℎ represents the bandwidth, *K*(·) denotes the kernel equation, and $x_{j}$ refers to the point near $x_{i}$ .

In essence, KM serves as a “local constant estimator.” However, LLRM offers a solution to the “boundary problem” while also proving to be more efficient and versatile across various data types than KM (53). The specific equation for LLRM is as follows:


(11) $$\begin{array}{c} \min\\ \left|a_{0},b_{0}\right| \end{array}\overset{n}{\underset{i=1}{\sum }}K\left[x_{i}-x_{0}/h\right]{\left[y_{i}-a_{0}-b_{0}\left(x_{i}-x_{0}\right)\right]}^{2}$$

Let us consider a scenario where “*h*” represents the broadband, and “*K*(.)” symbolizes the kernel function. When examining the observation value “*y_j_*” corresponding to the NNM “ $x_{0}$ ,” we assume that “*m(x)*” denotes a linear function concerning the NNM “ $x_{0}$ .” In Equation 11, the linear function can be expressed as ${"}^{m\left(x\right)}=a_{0}-b_{0}\left(x_{i}-x_{0}\right){,}^{"}$ where ${"}^{{a_{0}}^{"}}$ represents the constant term, and $b_{0}$ stands for the coefficients of $\left(x_{i}-x_{0}\right).^{"}$

In the next step, using the matched samples gathered previously, we proceeded to compare the average disparity in crop yield between growers in the treatment and those in the control group. The concept of the “average treatment effect among treated persons” (ATT) is formulated as follows Equation 12:


(12) $$ATT=E\left[\left(Y_{1}-Y_{0}\right)|D=1\right]=E\left\{E\left[\left(Y_{1}-Y_{0}\right)|D=1\right],\;P\;\left(X\right)\right\}$$

Here, 𝐷 represents the treatment variable taking values of 0 and 1, where *D* = 1 denotes the treatment group (farmers using the MI) and *D* = 0 indicates the control group (farmers not using the MI). *P*(*X*) signifies the propensity score value, while $Y_{1}$ and $Y_{0}$ are the estimated outcomes of wheat production for growers utilizing and not utilizing the MI, respectively.

## 4 Result and discussion

### 4.1 Descriptive statistics

The statistical attributes of the sample outlined in Table 2 show that the average age of growers utilizing MI stands at 48.50 years, which is lower than that of non-users, indicating a younger demographic among MI users. The average schooling of MI-using farmers is 3.12, surpassing that of non-users at 2.72, signaling a trend towards higher educational attainment among MI users, typically at or above the junior high school level. Furthermore, MI-using farmers exhibit a higher risk preference compared to non-users. With an average farm size of 1.90 hectares, MI users operate on larger scales than their non-MI counterparts, suggesting a preference for MI integration in production processes among farmers with larger holdings. Additionally, the number of plots owned by MI users is 4.30, which is inferior to the 5.39 plots owned by non-users. Finally, villages with MI users exhibit a higher level of economic development compared to those without MI users.

**Table 2:** Descriptive statistics.

Variable name	All	MI use (Not-use MI)	Difference
Wheat production (kg/ha)	7390.58	8080.20 (7155.10)	925.10^***^
Age	52.79	48.50 (53.50)	−5.043^***^
Education	2.72	3.12 (2.69)	0.430^***^
Plots	5.30	4.30 (5.39)	−1.135
Farm size	0.61	0.71 (0.59)	0.119^***^
Non-agricultural income	0.60	0.63 (0.59)	0.034
Livestock	1.90	1.80 (1.60)	0.020
Train	0.19	0.22 (0.18)	0.038
Extension	6.39	6.90 (6.31)	0.664^***^
Health	1.42	1.37 (1.43)	−0.065
Machinery	6.38	6.95 (6.27)	0.677^***^
Irrigation	4.79	4.55 (4.84)	−0.295
Seed	6.67	6.71 (6.66)	0.048
Fertilizer	7.68	7.82 (7.65)	0.167^***^
Pesticide	5.77	5.47 (5.83)	−0.361^***^
Risk preference	1.40	1.51 (1.38)	0.130^***^
Market distance	6.68	6.73 (6.69)	0.040
Economic development level	3.29	3.47 (3.26)	0.215^***^
Poor village	0.26	0.29 (0.25)	0.043

*** Is significant at the 1% level.

### 4.2 Influence of MI adoption on crop production: linear regression model

Current research employs the variance inflation factor to assess multicollinearity, revealing a value of 1.36, well below the threshold of 10, thus suggesting no significant multicollinearity issues. Additionally, robust standard error techniques are incorporated in regression analysis to address heterogeneity (54). Generally, the regression outcomes in Table 3 suggest a relatively suitable appropriate for the model. MI usage significantly affects crop production, with MI users increasing wheat yield per hectare by 1,059 kilograms, a 13.30% increase compared to non-users. From Models 2–5, it is evident that the influence of MI usage on farmers’ crop productivity varies. The coefficient for the level of education variables is optimistic and statistically substantial at the level of 1%, indicating that for every unit increase in education level, growers’ production per hectare increases by 25.30 kilograms. Conversely, the coefficient for the risk preference is adverse and statistically substantial at a 1% level, suggesting that as risk levels grow, wheat yield per hectare decreases. Specifically, an increase of one level in risk preference leads to a decrease of 655.5 kilograms per hectare in wheat production. Furthermore, for every unit boost in the percentage of non-agricultural revenue, wheat production per hectare declines by 925 kilograms. A higher proportion of non-agricultural income implies less focus on agriculture and production inputs, consequently resulting in a decrease in crop production (55).

**Table 3:** Shows the findings of the mediating method.

Variables	Wheat Yield per ha									
	Models									
	1		2		3		4		5	
MI-usage	1,059^***^	164.5	959.6^***^	170.3	794.7^***^	174.2	945.9^***^	199.0	958.9***	198.5
Age	7.714	6.959	10.81	6.992	6.815	7.056	3.266	7.400	-	-
Education	260.3^***^	80.00	296.1^***^	80.66	270.8^***^	81.51	265.1^***^	84.15	-	-
Plots	−47.02^***^	13.49	−47.49^***^	13.77	−47.55^***^	12.83	-	-	-	-
Farm size	810.2^***^	164.5	874.7^***^	166.4	966.6^***^	164.6	-	-	-	-
Non-Agri-income	−925.0^***^	220.4	−874.8^***^	224.3	−1,077^***^	230.9	-	-	-	-
Livestock	−40.01^***^	10.49	−40.49^***^	10.77	−40.55^***^	10.83	-	-		-
Train	−72.71	187.4	135.4	185.8	118.9	177.6	149.1	190.5	-	-
Extension	20.28	90.69	−40.96	101.2	−20.91	100.5	−100.6	100.4	-	-
Health	25.30	99.69	−48.96	100.2	−26.91	102.5	−106.6	105.4	-	-
Machinery	−132.9^***^	39.19	−150.4^***^	39.65	-	-	-	-	-	-
Irrigation	102.1^***^	26.36	114.3^***^	26.67	-	-	-	-	-	-
Seed	−180.8	137.9	−156.7	140.9	-	-	-	-	-	-
Fertilizer	252.2 **	103.3	314.6^***^	106.7	-	-	-	-	-	-
Pesticide	151.9^***^	46.75	155.6^***^	47.67	-	-	-	-	-	-
Risk preference	−655.5^***^	116.9	−667.1^***^	119.3	−639.2^***^	121.0	−688.7^***^	123.9	-	-
Economic development level	−291.0^***^	90.44	-	-	-	-	-	-	-	-
Poor village	−777.6^***^	175.2	-	-	-	-	-	-	-	-
Eastern reference group										
Middle Region	−1,288^***^	162.4	−1,410^***^	164.2	−1,450^***^	164.7	−1,558^***^	163.8	−1,525^***^	160.0
Western Region	418.6^*^	227.3	224.3	234.7	310.3	221.0	699.0^***^	228.5	546.5^**^	233.4
Northeast Region	638.6^***^	191.7	524.6^***^	196.6	320.0 ^*^	190.6	1,195^***^	174.6	1,032^***^	169.6
Constant	7,348^***^	1,218	5,495^***^	1,134	7,467^***^	588.0	7,442^***^	562.9	7,317^***^	114.2
Pseudo R^2^	0.285		0.259		0.223		0.145		0.115	

Significance levels are denoted as ^***^, ^**^, and ^*^ to signify statistical significance at the 1, 5, and 10% levels.

The coefficient for farm size is optimistic and substantial at the 1% level. Although Pakistan’s farms have not reached the scale of economies of crop production yet, increasing farm size still correlates with higher crop yields. This means that for every 1% rise in farm size, crop production per hectare rises by 810.2 kilograms (56). Conversely, the plot number has an adverse on crop production and is statistically substantial. This indicates that for every additional plot, wheat production per hectare decreases by 2.519 kilograms. The coefficient for seed cost is adverse and statistically substantial at the 1% level, suggesting that for every 1% rise in seed cost, production per hectare decreases by 47.02 kilograms. This may be due to excessive seed input leading to decreased production. The positive coefficient on pesticide expenses is statistically substantial at the 1% level. For every 1% increase in pesticide input, production per hectare increases by 9.795 kilograms, reflecting the ongoing reliance of agricultural production on pesticides.

The coefficient associated with fertilizer expenses shows statistical significance at the 5% level, with a positive direction. With every 1% rise in fertilizer input, crop yield per hectare rises by 20.46 kilograms, highlighting the crucial role of fertilizers in grain production (57). The coefficient for irrigation system inputs is optimistic and significant at 1% level, indicating that with every 1% increase in irrigation input, crop production per hectare increases by 102.1 kilograms, emphasizing the importance of water resources security in crops (58). The coefficient for technology cost is negative and statistically substantial at the level of one parent, signifying that with every 1% increase in machinery cost, wheat production per hectare decreases by 132.9 kilograms. This might occur due to improved machinery costs reducing other production inputs, thereby lowering wheat production per hectare. Compared to non-poverty-stricken villages, production per hectare in poverty-stricken villages decreases by 777.6 kilograms, possibly due to credit constraints limiting farmers’ investment in agricultural production. Additionally, the economic development of the village where growers reside also negatively impacts crop production, as confirmed by significance tests. Specifically, a decrease of one level in economic development leads to a decrease of 291 kilograms in wheat yield per hectare.

### 4.3 The PSM findings

The MI use in crop production does not pose an endogenous causality issue; rather, in this study, the endogeneity of the independent variable arises more from the self-selection bias within the sample. To address this self-selection issue, the PSM technique is employed and outcomes are presented in Table 4. Given the various matching methods available within PSM, to ensure robustness, matching techniques such as NNM, KM, LLRM, and RM are utilized to attain the ATT for MI-utilizing and non-utilizing growers (treatment and control groups). Existing outcomes indicate that the ATT constant is optimistic and statistically substantial at 1% levels; the average ATT across four different approaches is 1041.53 kilograms per hectare. Contrasted to the counterfactual scenario of non-MI-using growers, MI-using farmers experience a 12.80% increase in wheat production per hectare. This consequence aligns closely with the ordinary least squares technique, confirming the robustness of the study findings.

**Table 4:** MI adoption on crop production: (PSM outcomes).

Matching techniques	Treatment [Control]	ATT	S.E.	T Value
NNM	8250.10 [7160.05]	1077.08^***^	255.28	4.07
KM	8250.10 [7231.24]	1021.89^***^	233.50	4.4
LLRM	8250.10 [7210.57]	1027.55^***^	323.45	3.15
RM	8250.10 [7224.48]	1007.59^***^	233.60	4.15

S.E. shows standard errors; ^***^Indicates significance at the 1% level.

Balance testing assesses the effectiveness of matching in ensuring well-balanced data. Post-matching, there is a noticeable reduction in bias across most variables, with the majority of T-tests yielding non-significant results. This lack of rejection of the initial hypothesis, which posits no substantial variances between the treatment and control group, underscores the suitability of employing PSM. This analysis indicates minimal systematic discrepancies between these two groups, as presented in Table 5. Comparing the post-matching outcomes with the pre-matching ones reveals a significant decrease in the standardized deviations of most variables. Notably, only the deviation related to seed cost demonstrates an increase, yet this does not compromise the robustness of the PSM findings. This indicates that the vast majority of observations align within a common range, with PSM.

**Table 5:** Evaluating balance in PSM: Un-matching (U.M.) and Matching (M.).

Variables name	Treated		Control		Bias (%)	Reduce Bias	T Test	*p* > t
	U.M.	M.	U.M.	M.				
Age	48.54	48.84	53.59^***^	46.99	−48.8	82.8	−5.94	0.000
Education	3.13	3.07	2.70^***^	3.15	46.8	81.6	6.10	0.000
Plot	4.28	4.34	5.41	4.47	−15.6	88.5	−1.62	0.106
Farm size	0.70	0.70	0.60^***^	0.70	18.2	98	2.59	0.010
Non-Agri-income	0.62	0.61	0.58	0.61	9.3	80.7	1.23	0.217
Livestock	3.15	3.09	2.72^***^	3.17	46.10	81.8	6.12	0.000
Training	0.21	0.18	0.17	0.17	9.4	54.3	1.25	0.210
Extension	7.94	7.91	7.26^***^	7.89	32	96.6	3.71	0.000
Health	1.36	1.36	1.42	1.31	−10.6	20.1	−1.33	0.185
Machinery	6.94	6.91	6.26^***^	6.89	32	96.6	3.71	0.000
Irrigation	4.54	4.64	4.83	4.5	−09	96.5	−1.33	0.184
Seed	6.70	6.71	6.65	6.79	9.5	−74.9	1.08	0.282
Fertilizer	7.81	7.80	7.64^***^	7.80	22.6	97.6	2.9	0.004
Pesticide	5.46	5.60	5.82^***^	5.68	−22.0	77.9	−3.2	0.001
Economic development	3.46	3.47	3.25	3.38	24.6	56.1	3.24	0.001
Poor village	0.28	0.29	0.24	0.27	9.6	45.7	1.28	0.201
Risk preference	1.50	1.50	1.37^***^	1.57	20.3	42.9	2.74	0.006

*** Is significant at the level of 1%.

PSM technique is designed to mitigate the effect of the observable variable on outcomes, but it does not address potential biases stemming from unobservable factors. Rosenbaum (59) introduced boundary analysis as a method to assess the impact of these hidden variables on PSM outcomes, denoted by the parameter Г. A value of Г = 1 signifies a scenario without hidden bias, with higher values indicating greater hidden bias (60). To gauge the robustness of these findings, this research established a range of values for Г based on the wheat yield data from the current study. With 90% of sample households yielding less than 11222.61 kilograms per hectare, we established the range as Г*∈* [1.15]. Each Г value, calculated upper and lower bounds for the significance level, demonstrating that PSM results maintain consistency even in the presence of hidden biases. Particularly, when Г is ≤1.2, this outcome is substantial at the level of 1%; for values between 1.2 and 1.4, significance holds at the 5% level and for values at 1.45 and 1.5, significance returns to the 1% level. This underscores the reliability of PSM outcomes despite potential increases in hidden bias. Refer to Table 6 for a detailed summary of comprehensive research findings.

**Table 6:** Assessing the Rosenbaum-bound crop productivity offers insights for optimizing yields.

Γ	Sig+ (Sig−)	Γ	Sig+ (Sig−)
1.0.	0.000142 (0.000142)	1.30	0.018722 (7.90 × 10^–8^)
1.05	0.000421 (0.000043)	1.35	0.031332 (2.10 × 10^–8^)
1.10	0.001101 (0.000013)	1.40	0.049385 (5.60 × 10^–9^)
1.15	0.002564 (3.70 × 10^–6^)	1.45	0.073802 (1.40 × 10^–9^)
1.20	0.005408 (1.10 × 10^–6^)	1.50	0.105186 (3.70 × 10^–10^)
1.25	0.010455 (2.90 × 10^–7^)	-	-

Sig + (Sig−) lower and upper bound significance levels.

### 4.4 The heterogeneity analysis

The outcomes of the heterogeneity investigation are revealed in Table 7 (where ATT represents the average ATT values for the matching approaches). Given the aging rural population in Pakistan, this study divides farmers into two groups based on the retirement age of 50 for men in Pakistan: those aged 50 and above, and those below 50. The findings reveal that for farmers below 50, the ATT value is optimistic and statically, substantial, indicating a significant effect of MI usage on crop production in this age group. MI-using farmers under 50 experience an increase of 1019.20 kilograms per hectare (13.50%) in wheat production compared to their non-MI-using counterparts. One possible explanation for this is that farmers under 50 possess stronger abilities to access and process information compared to those aged 50 and above, thus significantly enhancing their managerial skills (61). MI usage does not significantly affect crop production for farmers aged 50 and above. This could be because elderly farmers are less inclined to use computers and smartphones to access information via the Internet of Things, and farmers are improbable to incorporate these latest technologies into sustainable agriculture systems.

**Table 7:** Farmers’ attributes and heterogeneity assessment [Matching techniques (MT)].

MT	Age		Education		Farm Size		Economic Development Level	
	<50	≥50	Low	High	Small-scale farmers	Large-scale farmers	Undeveloped Village	Well-developed Village
NNM	1035.80^***^ (277.95)	229.89 (1121.81)	1281.90^***^ (327.52)	1082.65** (482.68)	71.25 (237.49)	2022.84^***^ (611.46)	198.61 (382.79)	728.10 * (425.69)
KM	1018.57^***^ (253.47)	318.11 (1086.73)	1121.03^***^ (298.79)	974.01 **(475.66)	155.61 (205.93)	1709.57^***^ (604.22)	255.49(367.49)	773.50** (383.23)
RM	1027.39^***^ (253.19)	320.03 (1086.29)	1119.97^***^ (298.66)	976.12 **(475.29)	140.91 (205.47)	1713.18^***^ (589.85)	251.24 (367.89)	778.99 ** (385.14)
LLRM	1001.96^***^ (331.61)	181.85 (1399.20)	1080.19^***^ (421.08)	1017.02 *(580.43)	109.95 (324.00)	1718.18 ** (734.57)	234.33 (556.73)	903.06* (479.61)
ATT	1019.20	262.47	1149.88	1011.50	119.43	1789.90	234.92	795.80

The significance levels are denoted as ^***^(1%), ^**^(5%), ^*^(10%).

Given the prevalent low educational attainment in rural locations, this study categorizes farmers with high school education or lower as low-educated, and those with high school education or higher as high-educated. The findings reveal that low-educated farmers who use the MI experience an increase of 1149.88 kilograms (15.27%) per hectare in wheat production compared to non-users. Similarly, highly educated farmers who use the MI witness an increase of 13.50% per hectare in yield compared to their non-using counterparts. One potential explanation is that growers with lower schooling typically have limited knowledge abilities, and MI usage could pointedly enhance their capability to access and process knowledge (62). According to Zheng et al. (63), the study categorizes farmers based on their farm sizes: those with 1.33 ha and below are deemed growers, while those with above 1.33 ha are considered large-scale growers. The findings reveal that large-scale growers witness a significant positive impact when utilizing MI. Specifically, compared to non-MI users, large-scale farmers employing MI increase their wheat yield per hectare by23.81%. Agriculture constitutes the primary income source for these larger farmers. Utilizing MI can help them maximize income and enhance the distribution of farming system inputs. The adoption of ICT has notably influenced large-scale food production among growers (64).

In villages classified as economically developed, farmers benefit from a positive and statistically significant ATT from MI usage. The impact of MI on crop production is particularly pronounced in these developed villages. Farmers who use MI in these areas experience an increase of 10.50% per hectare in wheat yield compared to their counterparts who do not use MI. One potential explanation is that economically prosperous villages tend to offer improved agricultural production environments, enabling growers to access useful farming information via MI. Consequently, they can enhance productivity inputs and crop production (65). Conversely, in economically disadvantaged villages, the decision to use wheat flour may not significantly impact wheat yields. This observation indirectly imitates the Matthew effect of MI usage on wheat production. Heterogeneity examination additionally approves the facilitating role of MI usage in growers’ crop production. Looking at the heterogeneity outcomes of growers, there are significant changes in crop production, especially among large-scale farmers, in terms of MI usage among growers’ ages, schooling, farm sizes, and village economics progress levels. Also, the matching outcomes of various approaches vary.

## 5 Conclusions and policy implications

### 5.1 Conclusion

The current study analyzes data from 660 crop farmers across two provinces in Pakistan, revealing a significant correlation between the use of MI and increased wheat yields. Farmers employing MI experience a remarkable 13.30% increase in wheat yield compared to those who do not use this technology. PSM analysis supports these findings, indicating a robust yield increase of 12.80%. The impact of MI on crop production varies across different farmer demographics. Notably, younger, less educated farmers with larger farms and those residing in economically developed villages see even greater yield improvements of 13.50, 15.27, 23.80, and 10.50%, respectively, compared to their non-MI-using peers. Furthermore, this research suggests that enhancing education system for growers, increasing farm size, and optimizing fertilizer use could be essential strategies for further boosting wheat yields. The article concludes that MI usage has a significant positive effect on crop production, emphasizing its potential benefits for farmers.

### 5.2 Policy implications

This research highlights the critical role of integrating ICT into Pakistan’s agricultural sector. The study emphasizes three core strategies for driving this integration. First, government intervention is crucial to incentivize farmers to adopt MI technologies for accessing agricultural data. Despite the potential to enhance productivity, MI adoption remains limited, necessitating efforts to boost digital literacy and increase awareness of online agricultural tools. Second, policy development must account for the varying educational levels among farmers. Those with less formal education often struggle to access and interpret agricultural information, making MI an essential tool for bridging these knowledge gaps. Tailored training initiatives could help farmers overcome digital literacy challenges, ensuring more equitable access to valuable resources. Lastly, the study recommends promoting farm consolidation as a means to enhance production efficiency. Larger farms tend to experience greater benefits from MI use, resulting in higher productivity. To support this, the government could simplify the land transfer process, fostering the growth of large-scale agricultural enterprises.

### 5.3 Existing research limitations

This study have some limitations. Firstly, the usage of PSM to measure the influence of MI usage on wheat yield relies solely on observable variables, neglecting any unobservable factors. Although the presence of unobservable variables appears minimal in this study, their exclusion could still introduce bias. Secondly, the definition of the “ICT usage such as MI” variable might be somewhat simplistic. It merely indicates whether farmers use the MI without considering the quantity or diversity of agricultural information accessed. This oversight could potentially influence the research outcomes. Research by Ma et al. (56) and Nie et al. (57) indicates that farmers predominantly utilize computers and mobile to access the internet technology. A promising avenue for future research could explore how different methods and types of internet information achievement affect growers’ food production.

## Data Availability

The raw data supporting the conclusions of this article will be made available by the authors, without undue reservation.
